# A dual-isotope approach to allow conclusive partitioning between three sources

**DOI:** 10.1038/ncomms9708

**Published:** 2015-11-04

**Authors:** Thea Whitman, Johannes Lehmann

**Affiliations:** 1 Soil and Crop Sciences, School of Integrative Plant Science, Cornell University, Ithaca, New York 14853, USA; 2 Atkinson Center for a Sustainable Future, Cornell University, Ithaca, New York 14853, USA

## Abstract

Stable isotopes have proved to be a transformative tool; their application to distinguish between two sources in a mixture has been a cornerstone of biogeochemical research. However, quantitatively partitioning systems using two stable isotopes (for example, ^13^C and ^12^C) has been largely limited to only two sources, and systems of interest often have more than two components, with interactive effects. Here we introduce a dual-isotope approach to allow conclusive partitioning between three sources, using only two stable isotopes. We demonstrate this approach by partitioning soil CO_2_ emissions derived from microbial mineralization of soil organic carbon (SOC), added pyrogenic organic matter (PyOM) and root respiration. We find that SOC mineralization in the presence of roots is 23% higher (*P*<0.05) when PyOM is also present. Being able to discern three sources with two isotopes will be of great value not only in biogeochemical research, but may also expand hitherto untapped methodologies in diverse fields.

## Abstract (web summary)

Stable isotopes are a useful tool for distinguishing two sources in a mixture, but frequently systems have more than two components. Here, the authors propose a new approach to allow conclusive partitioning between three sources, still using only two stable isotopes, looking at soil CO_2_ emissions.

## Graphical abstract

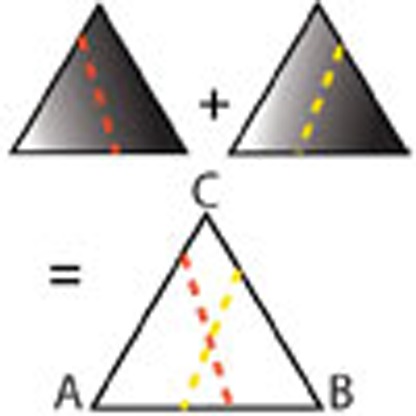

Stable isotopes have proved a transformative tool in the geosciences and beyond, enabling studies ranging from geochemical cycling across scales^1^,^2^,^3^, ecological food web and resource partitioning^4^,^5^, plant and microbial nutrient and water use^6^,^7^ and forensics^8^,^9^. Isotopic partitioning has been used by biogeochemists for decades to distinguish between two different sources of an element (for example, soil versus plant C or N), underpinning much of what we know about the global C and N cycles^1^,^6^,^10^. However, conclusively partitioning a system using two stable isotopes (for example, ^13^C and ^12^C) has up to now been largely limited to only two sources (except see ref. 11), and systems of interest often have more than two components, with interactive effects.

Briefly, if the isotopic makeup of a mixture is known, and the two sources have different isotopic makeups, it is possible to partition the combined pool between the two sources (here, A and B):


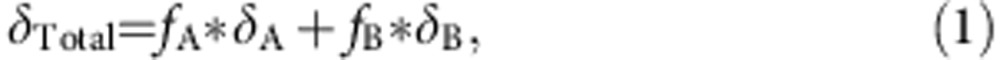


where *δ*_X_ is the known isotopic ratio of the individual sources (*δ*_A_ or *δ*_B_) or their combined total (*δ*_Total_), and *f*_A_ and *f*_B_ represent the fraction of total pool made up by A and B, respectively^10^. Because we also know


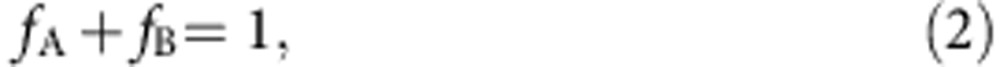


we can combine the two equations and solve for the two fractions. However, if a third source, C, is introduced, we now have three unknowns but still only two equations:


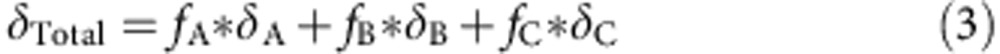



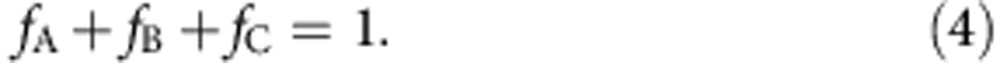


Thus, the problem is underdetermined^12^ and there is now a range of possible solutions (illustrated in Fig. 1a).

Previous approaches have addressed this problem in a number of ways (Table 1). Important advances have included the development of models that consider multiple sources^12^, and Bayesian methods including multiple sources along with probability estimates for the subsequent partitioning^13^,^14^. These models are currently our best tools for evaluating mathematically underdetermined systems, where we have more unknown variables than equations. However, all existing approaches have the drawbacks that either (i) the partitioning is not conclusive (that is, a range or distribution of solutions is calculated) or (ii) the systems to which they apply are limited because for some sources (for example, soil), natural differences in isotopic abundance are also associated with other physical, chemical or biological differences, and it is not possible to evenly introduce an isotopic label (Table 1). Here, we present a solution to this problem by introducing a three-source dual-isotope approach. By adding an additional treatment that is identical in every way to the first, except for that it has a second and different isotopic signature for only one of the three sources, we convert equation (3) into two equations:


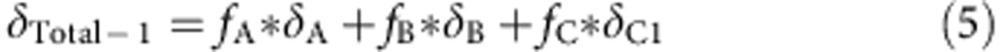



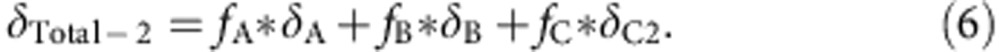


When equations (5) and (6) are combined with equation (4), we now have three different equations, and three unknowns, and are thus able to conclusively partition the pool into its three sources (illustrated in Fig. 1b). One example of solving the system of equations (4, 5, 6) for the system's three constituent fractions is:


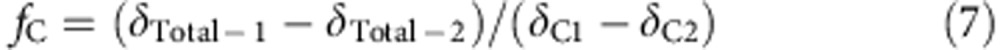



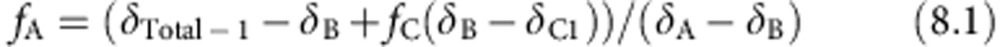


or


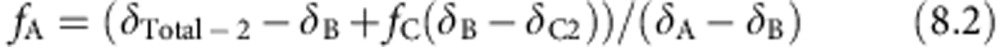



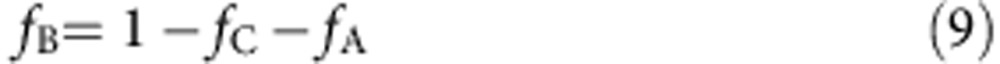


To estimate variance (*σ*^2^) for each of these fractions, we used first-order Taylor series approximations^15^ and a matrix-fitting approach, detailed in Supplementary Note 2. The R scripts used for these calculations were designed to be easily applied to other users' data sets, and are included as Supplementary Data sets 1–3.

To demonstrate this three-source dual-isotope approach, we applied it in an environmental setting in order to partition CO_2_ emissions derived from microbial mineralization of native soil organic carbon (SOC), of added pyrogenic organic matter (PyOM) and from root respiration. Whether PyOM is produced naturally in fires^16^ or intentionally for C management and/or as an agricultural amendment^17^,^18^, it is essential to understand the effects of its production and addition to soils on the C cycle^19^. We know that PyOM additions to soil can have significant effects on plant growth and crop yields (for example, see ref. 20 for details). In addition, it has been established that PyOM additions can affect SOC dynamics in a wide variety of ways, sometimes increasing and sometimes decreasing C mineralization^21^,^22^. Given these known two-way effects, there are strong expectations that complex three-way C cycling interactions could occur in three-source systems with PyOM, plants and soils. For example, there is a wide range of mechanisms by which plant roots can affect SOC dynamics^2^. In systems where PyOM additions alter plant growth or root exudate patterns, these plant root–SOC mineralization interactions may change. Despite this prediction, such effects have been scarcely considered, with Whitman *et al*.^23^, Major *et al*.^24^ and Slavich *et al*.^25^ being the only exceptions of which we are currently aware. These three studies support the prediction of complex three-way interactions between soils, plant roots and PyOM, but none of them was designed in such a way that the three C sources could conclusively be partitioned. A solution to this methodological problem, such as that which we present here, is sorely needed in order to truly understand the effects of PyOM on the C cycle, as well as for numerous other analogous three-source systems. Using this three-part partitioning approach, we find that SOC mineralization in the presence of roots was 23% higher (*P*<0.05) when PyOM was also present. This confirms that there may be important three-way interactions between the C dynamics of plant roots, PyOM and SOC, and illustrates this approach as a promising method for other three-part systems.

## Results

### Partitioning of CO_2_ emissions

**Figure 2:**
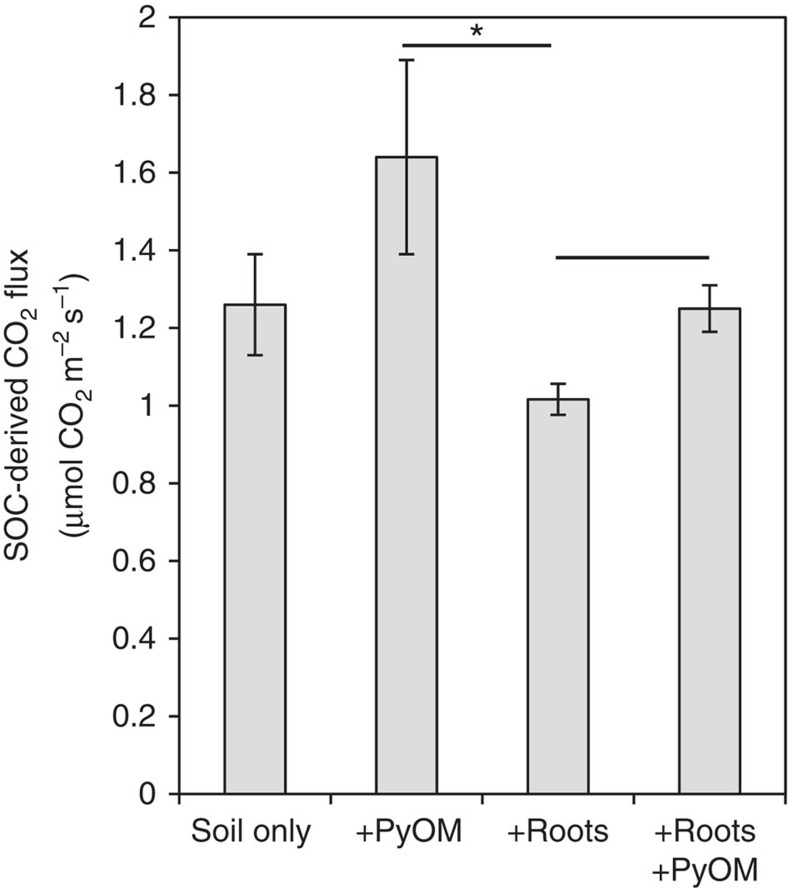
CO_2_ fluxes from soil organic carbon. Data shown for one-, two- and three-source systems for one time point (day 66) of the field trial. Error bars±s.e.m., *n*=7. *A significant difference (*P*<0.05) in SOC-derived fluxes from plots with roots versus plots with PyOM and roots present. ·Difference (*P*<0.08) in SOC-derived fluxes from plots with roots versus plots with PyOM.

Using the two- and three-source partitioning approaches described above, we determined that, compared to plots with roots alone, SOC mineralization increased by 61% in plots with PyOM only (*P*<0.08) and increased by 23% in plots with PyOM in addition to roots (*P*<0.05; Fig. 2). This effect would have remained undetectable without using the tested three-source partitioning and has therefore to our knowledge not been unambiguously quantified previously.

### Sources of variability

Variability is a major constraint in detecting differences, especially in field studies, as utilized here. The trends towards decreased PyOM mineralization in the presence of roots (Supplementary Table 3), and higher root-derived CO_2_ in the presence of PyOM (Supplementary Table 3) could be important, and the utility of this method depends on our ability to detect them. For example, with our current data set, a 41% decrease in PyOM emissions was not significant at *P*<0.05—we would have required 20 replicates to detect such differences at these levels of variance. Although higher than standard for many field trials, 20 replicates are not necessarily impractical. To help address this issue, we aimed to determine which had a greater effect on our findings: variability in flux rates, or variability in the measured *δ*^13^C values of total fluxes and their constituent sources (Supplementary Note 3 and Supplementary Table 3). We see that the main source of variability in the final partitioned fluxes is primarily due to variability in the measured fluxes, rather than within the isotopic compositions of sources. This highlights that, as with other isotopic methods, preliminary trials to determine expected variability, many replicates and/or attempts to limit heterogeneity will be important in optimizing experiments using this method.

### Possible effects of isotopic fractionation

We investigated the effects of possible isotopic fractionation, which is known to occur during PyOM mineralization^23^, plant metabolism^26^,^27^ and SOC mineralization^28^,^29^ (described in Supplementary Note 4 and Supplementary Table 4) and is common in both biotic and abiotic systems. Intriguingly, we find that the calculation of the dual-labelled source (here, PyOM) is relatively insensitive to effects of fractionation, so long as fractionation is similar between the two sources. This occurs because equation 7 depends only on the difference between the two sources.

## Discussion

Although increases in SOC mineralization in the presence of roots have been observed more often than decreases for a variety of reasons^2^, decreases are sometimes attributed to fast-growing microbes shifting from SOC to root-derived C sources in soils with limited N levels^2^. If preferential mineralization of root-derived C was reducing total SOC mineralization, the presence of PyOM may have counteracted the effect of roots on SOM mineralization by adsorbing root exudates and protecting them from mineralization by microorganisms. Strong adsorption of dissolved organic C to PyOM in soils is a well-established phenomenon^30^ and co-location of SOC on PyOM surfaces has been demonstrated by nanoscale secondary ion mass spectrometry and related to lower mineralization induced by PyOM^31^. In addition, roots have been observed to grow in pores of PyOM^32^, making exudate deposition on PyOM particles plausible. Although it is conceivable that highly porous PyOM could have counteracted inhibition due to decreased soil moisture in the presence of roots, this explanation is not likely, as the measurements took place during a period with relatively high precipitation (Supplementary Fig. 2), and we did not find significant differences in moisture between plots at the end of the trial. Direct effects of PyOM on SOC (for example, increased soil pH from 6.0 to 6.75; *P*<0.06) are also an unlikely explanation in this study, as PyOM did not significantly increase SOC mineralization without the presence of plants. These measurements illustrate the value of this new three-source partitioning approach: conclusively and quantitatively partitioning emissions in the three-source system to detect important two- or three-way interactions would not be possible with a conventional approach using two isotopes. It is easy to see that being able to detect such effects will have important real-world consequences, and without approaches such as that described here, we will not be able to conclusively detect these effects. Our three-source dual-isotope approach is appealing in that it does not require that multiple components have ‘identical' isotopic signatures, which can be difficult to achieve for most biogeochemically relevant components and is nearly impossible for native soil organic matter or two different plants, as is the case in many other systems. In addition, our approach requires only two ‘treatments'. For future applications, we would make a few recommendations: because of the high error often associated with isotopic partitioning methods due to the high multi-level heterogeneity of natural systems, designing experiments with large numbers of replicates is ideal. In addition, although we created the different ^13^C signatures in the added PyOM by combining different ratios of highly enriched and natural abundance PyOM, generating the two sets of isotopically labelled PyOM materials by growing them in differently enriched atmospheres would result in a more uniform label. This would reduce sample heterogeneity, which is particularly important for analyses that require only very small masses of material or spatial investigations (Supplementary Table 2). However, as two different isotope ratios are always needed for the same material, mixing is more practical, cost effective and straightforward to achieve clearly different isotope ratios. Finally, as with other isotope studies, it is advisable to consider what the predicted relative contribution of the different sources will be, and ideally design the experiment so that those sources that will contribute the least to the total have the strongest isotopic label to improve detection limits. In addition, we note that the underlying approach we present here (to resolve what was a previously underdetermined system by adding a doubly labelled component) could be extended to systems with even more than three sources. Although some of the advantages of our approach (only one component needs to be labelled, and there is only one additional treatment) would be lost as more sources are added, there may be multi-component systems in which it could be useful.

**Figure 3:**
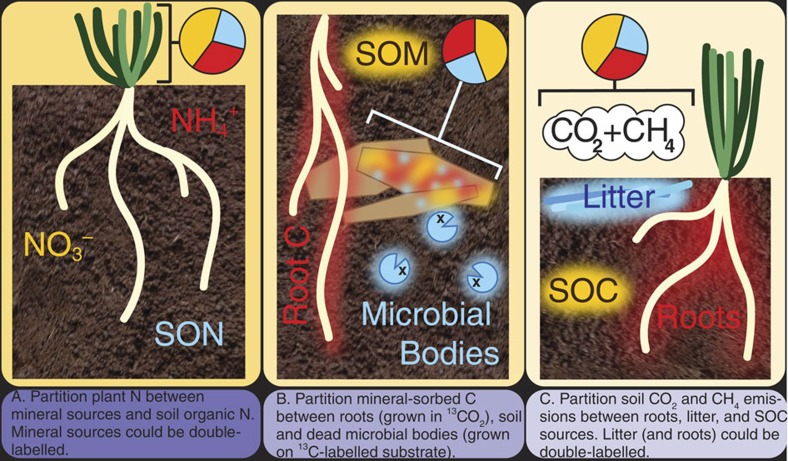
Suggested applications of the three-source dual-isotope partitioning method. Three examples for possible use of the method. Red, yellow and blue indicate the different sources that are being partitioned (depicted as pie charts).

We highlight this new partitioning approach because we believe it is a powerful tool to add to our isotopic toolkit for biogeochemical studies (Table 1), and is likely to catalyse inquiry that was not possible with the traditional two-isotope method. The list of possible applications is long (Fig. 3), and includes partitioning root contributions (respiration versus exudates and root turnover) to soil CO_2_ fluxes, stable isotope probing of DNA, plant, animal or microbial nutrient uptake, and distinguishing sources of greenhouse gases, among others. For example, the approach could be used to partition N sources in a plant, by providing an organic N source, NO_3_ and NH_4_, with one of the mineral sources being somewhat enriched in ^15^N, and the other enriched in ^15^N at two different levels, as described here. The three-source dual-isotope approach can be applied to a wide range of three-source systems—anywhere where one component could be produced with two different isotopic labels, with the remaining two components distinguished by natural abundance isotope differences or also labelled. Being able to discern three sources with two isotopes will be of great value not only in biogeochemical research, but may also expand hitherto untapped methodological opportunities in diverse fields.

## Methods

### Experimental design

A 2 × 2 factor field trial was conducted in eight replicates, with ^13^C-labelled PyOM additions and presence of plants as the two factors. In addition, a fifth treatment was added which also received PyOM amendments and plants, but where the PyOM had a higher ^13^C label. Two sets of corn plants (*Zea mays* (L.)) were grown, one in an enriched ^13^CO_2_ atmosphere growth chamber and the other in an ambient ^13^CO_2_ greenhouse. The labelled plants were grown in potting mix in a Percival AR-100L3 CO_2_-controlled growth chamber (Percival). The plants were exposed to cycles of 18 h light/6 h darkness. During light cycles, the atmosphere was maintained at 400 p.p.m. CO_2_, where CO_2_ was allowed to accumulate as a result of respiration during the dark cycle, and was then drawn down by photosynthesis during the next light cycle. This was done in order to reduce net respiratory losses of labelled ^13^CO_2_. Plants were pulse-labelled with 13 l of 99% ^13^CO_2_ at regular intervals over the course of their growth in order to produce an even label. Pulse labels were delivered by opening the ^13^CO_2_ cylinder to fill a balloon with ∼500 ml of ^13^CO_2_. The balloon remained attached to the cylinder so that the ^13^CO_2_ slowly diffused out of the balloon, delivering the pulse at a rate so that the total atmospheric concentration of CO_2_ was not affected. Plants in the growth chambers and the greenhouse were harvested just before they reached reproductive maturity and were oven-dried at 70 °C. The milled corn (<2 mm) was pyrolysed in a modified muffle furnace by ramping by 5 °C min^−1^ to 350 °C, then holding at 350 °C for 45 min, under Ar (properties are given in Supplementary Table 5). The ^13^C-labelled and natural abundance corn PyOM materials were mixed together at masses of 12.5 g labelled PyOM+187.5 g unlabelled PyOM for the lower label treatment and 31.35 g labelled PyOM+168.7 g unlabelled PyOM for the higher label treatment. Mixing was done in plot-level batches to ensure that each plot received exactly these proportions of labelled and unlabelled materials.

### Field site and soil description

The field site is located in Cornell's research fields in Mt. Pleasant, NY, and is a Mardin soil (Coarse-loamy, mixed, active, mesic Typic Fragiudept; properties of the soil are given in Supplementary Table 6). The soil has been historically planted to a potato, rye, clover rotation, for the past >30 years, but was kept in rye-clover rotation for the past 5 years, with one planting of sudangrass (*Sorghum bicolor* L. *x Sorghum sudanense* (Piper)) 3 years ago. The plot was sprayed with Roundup (glyphosate) herbicide in the fall, ploughed on 3 May 2013, and kept weed-free using hand-weeding and water-permeable landscape fabric through the summer until trial initiation. The trial initiation date was 16 August 2013 (day 0). Square plots (0.7 × 0.7 m^2^) were surrounded by 0.7-m-wide weed-free borders maintained by hand weeding between every plot and along the edges. Treatments were organized using a spatially balanced complete block design^33^. Thus, we did not consistently place the dual three-part treatments directly adjacent to each other, and so did not treat them as paired during statistical analyses. A total of 6.1 kg of soil were removed from the top layer of soil at each plot, combined with PyOM additions for the appropriate plots, and mixed in a V-mixer. Amended plots received 4.1 t ha^−1^ of PyOM. Mixed soils were then returned to their respective plots and evenly spread at the surface. Soil was gently tamped down using a flat piece of plywood. Soil respiration collars made from 194-mm diameter white polyvinylchloride (PVC) pipes were installed at the centre of the plots with the collar protruding 30 mm and reaching 30 mm into the ground. The lower portion of the collar had holes drilled in it to allow roots to penetrate the soil-amendment mixtures belowground. Sudangrass seeds (*Sorghum bicolor* L. *x Sorghum sudanense* (Piper)) were planted in six groups of three, evenly spaced around the centre of the plot and thinned to six plants 2 weeks after emergence. Plots were kept covered with water-permeable landscape fabric except during measurement until plant emergence, in order to keep weeds down. After plant emergence, plots were kept weed-free by hand-weeding multiple times a week.

### Soil CO_2_ flux and ^13^CO_2_ measurements

Soil CO_2_ flux was measured on day 66 using a LI-6400XT portable infra-red gas analyser with a 6400-09 soil CO_2_ flux chamber attachment (LI-COR). Three flux measurements were taken in succession for each plot and averaged. Samples were taken for ^13^CO_2_ analyses using modified static Iso-FD chambers^34^. Briefly, isotopic forced diffusion semi-static chambers were designed based on Nickerson *et al*.^34^ to allow for a simple estimate of the *δ*^13^C signature of soil CO_2_ fluxes. Chambers were machined out of aluminum and a Gore-Tex membrane was used to allow partial diffusion. Chambers were deployed in pairs, with one chamber top placed on the PVC soil collar, and the atmospheric reference chamber placed on a directly adjacent plugged PVC collar with the same volume as the chamber connected to the soil. The Gore-Tex material was sealed to the chamber with a rubber O-ring held tight with screws between the top and the sides of the metal chamber top. For each plot, collars were capped for 10–30 min with an adjacent atmospheric reference chamber capping a plugged collar. Samples of 20 ml air were then drawn from the sample and the reference chamber, and injected into 12.5 ml evacuated vials. Samples were analysed for *δ*^13^CO_2_ and [CO_2_] on a Thermo Scientific DELTA V isotope ratio mass spectrometer (Thermo Scientific) interfaced with a Gasbench II.

### Data processing and statistical analyses

All statistical analyses were performed in R^35^. We excluded flux data points that were more than 3 standard errors of the mean away from the mean, which resulted in *n*=7 for each treatment instead of *n*=8. We determined the isotopic signature of total emitted CO_2_ as per Nickerson and Risk^34^, and partitioned the two- and three-source systems using the equations described above. For the three-source system, we used two treatments that were identical except for the isotopic signature of the added PyOM (Supplementary Table 2). Detailed calculations are described in Supplementary Note 2 and Supplementary Data sets 1 and 2, where R scripts are provided. To attribute significant differences between fluxes, we used 95% confidence intervals where two given values do not overlap (*P*<0.05).

## Additional information

**How to cite this article:** Whitman, T. & Lehmann, J. A dual-isotope approach to allow conclusive partitioning between three sources. *Nat. Commun.* 6:8708 doi: 10.1038/ncomms9708 (2015).

## Supplementary Material

Supplementary Information Supplementary Figures 1-3, Supplementary Tables 1-6, Supplementary Notes 1-4 and Supplementary References.

Supplementary Data 1 R script for three-part partitioning of fractions and error calculation using Taylor series error propagation

Supplementary Data 2 R script for three-part partitioning of fractions and error calculation using matricies

Supplementary Data Data to be used as examples with the R scripts provided as Supplementary Datasets 1.R and 2.R.

## Figures and Tables

**Figure 1:**
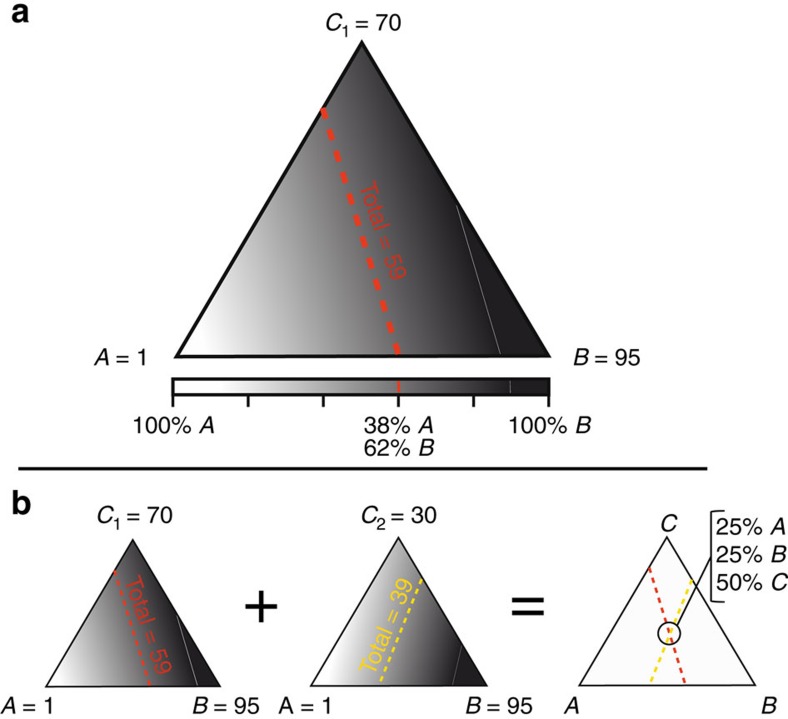
Concept of multiple isotopic partitioning solutions to a hypothetical three-source system. Shading corresponds to illustrative values representing the isotopic composition (in arbitrary units) of each end-member (A, B and C) or the combined isotopic composition for a given system. Dashed lines along a single combined isotopic composition represent possible partitioning solutions for the combined sources. (**a**) Given an isotopic composition (here, a shade of grey representing an isotope value of 59) for a two-source system (rectangle between A and B) there is only one solution (that is, 38% from A and 62% from B), but for the three-source system (triangle of A, B and C), there is a range of solutions, shown along the dashed red line, which does not allow the three sources to be conclusively quantified. (**b**) By designing a system with two treatments, where the third component is present in two different isotopic forms that are otherwise identical (two leftmost triangles, as indicated by different shades for C_1_ and C_2_), the range of solutions (dashed lines) will intersect at one point, which allows the total emissions to be partitioned conclusively into three parts (intersection of dashed lines in right triangle, indicating 25% A, 25% B and 50% C.

**Table 1:** Approaches to partitioning more than two sources*.

**Approach**	**Limitations**†	**Advantages**	**For example**
1. Additive approach: Include treatments without a given component, and then subtract the effects of the simpler system from the more complex system to estimate the effect of the component alone	Excludes the potential to detect any interactive effects and thus will not work for systems where components behave differently when isolated—that is, does not truly allow for three-source partitioning	Does not require additional treatments; likely least expensive method due to fewer isotopically labelled components	36,37
2. Modelling approach: Use modelling to calculate a range of possible partitions and their associated probabilities	Can only provide a range of solutions, which may not be optimal for some study questions	Provides probability distribution of solutions; explicitly accounts for natural underlying variation in isotopic composition of the sources	6,13,14
3. Multiple element approach: Use combinations of multiple elements (for example, C and/or N and/or O)	The range of applications is limited to systems where the different elements persist and/or cycle together	Does not require additional treatment; could provide additional insight due to multiple elements	8,38
4. Multiple isotope approach: Use two stable isotopes and a radioisotope (for example, ^12^C, ^13^C and ^14^C)	^14^C in soils is highly heterogeneous at natural abundance levels; enriched levels of ^14^C are highly regulated and generally cannot be used outside the laboratory; analyses can be expensive	Allows for conclusive three-source partitioning; highly enriched ^14^C could allow for high sensitivity	39
5. Combined sources approach: Collapse into what is effectively a two-source system, where all but one source (or groups of sources) have the same isotopic signature. To isolate individual components, the labelled source can be switched	Requires that at least two pairs of different sources are found or created with identical isotopic signatures without changing their other properties; some sources (particularly, soil) are extremely difficult to label evenly; even if the two sources have the same bulk isotopic signature, it is virtually impossible to find two sources that generate the same isotopic signal over time and space when they are controlled by different external factors (for example, soil CO_2_ emissions versus plant root respiration); likely requires isotopically labelled materials	In the right system, can allow conclusive three-source partitioning	40,41
6. Three-source dual-isotope approach: Use dual treatments with different isotope ratios for the same component	Likely requires isotopically labelled materials	Allows for conclusive three-source partitioning	This study

^*^Further examples, including a worked example of the additive approach, can be found in Supplementary Note 1, Supplementary Table 1 and Supplementary Fig. 1.

^†^All stable isotopic methods suffer from the limitation that isotopic fractionation may occur during biological and chemical transformations. Thus, identifying the appropriate end-members, or isotopic signatures, for the sources is essential.
