# Supplementary material for: A dual-isotope approach to allow conclusive partitioning between three sources
Source: Nat Commun. 2015 Nov 4;6:8708. doi: 10.1038/ncomms9708 (PMC4667633; doi:10.1038/ncomms9708)
Supplement: Supplementary Information — Supplementary Figures 1-3, Supplementary Tables 1-6, Supplementary Notes 1-4 and Supplementary References. [file ncomms9708-s1.pdf]

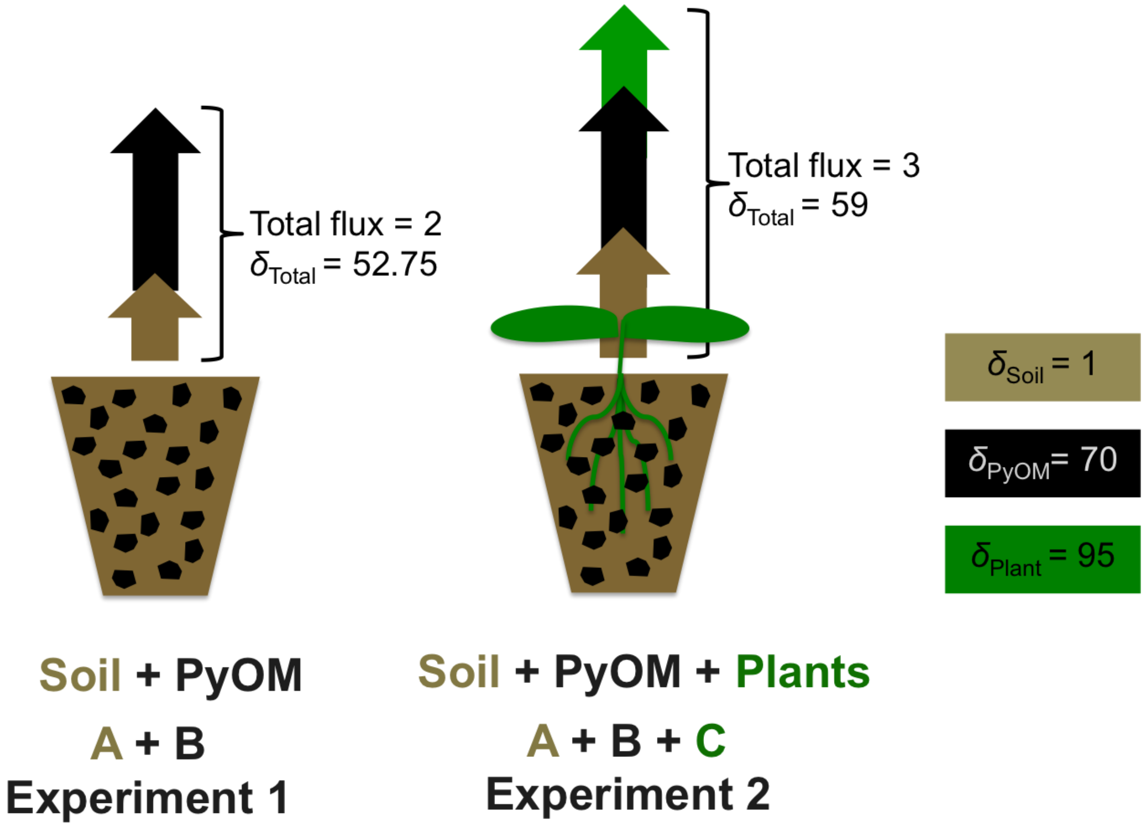

Supplementary Figure 1. Example used to demonstrate the additive approach. Arrows represent CO_2 flux, in µmol m^{-2} s^{-1}. In Experiment 1, the two sources, soil and PyOM, are conclusively partitioned. In Experiment 2, the assumption could be made that the soil and PyOM contribute the same amount (2 µmol m^{-2} s^{-1}) to the total flux as in Experiment 1, but this would be an error – interactions between the plants, soil, and PyOM have altered their contribution.

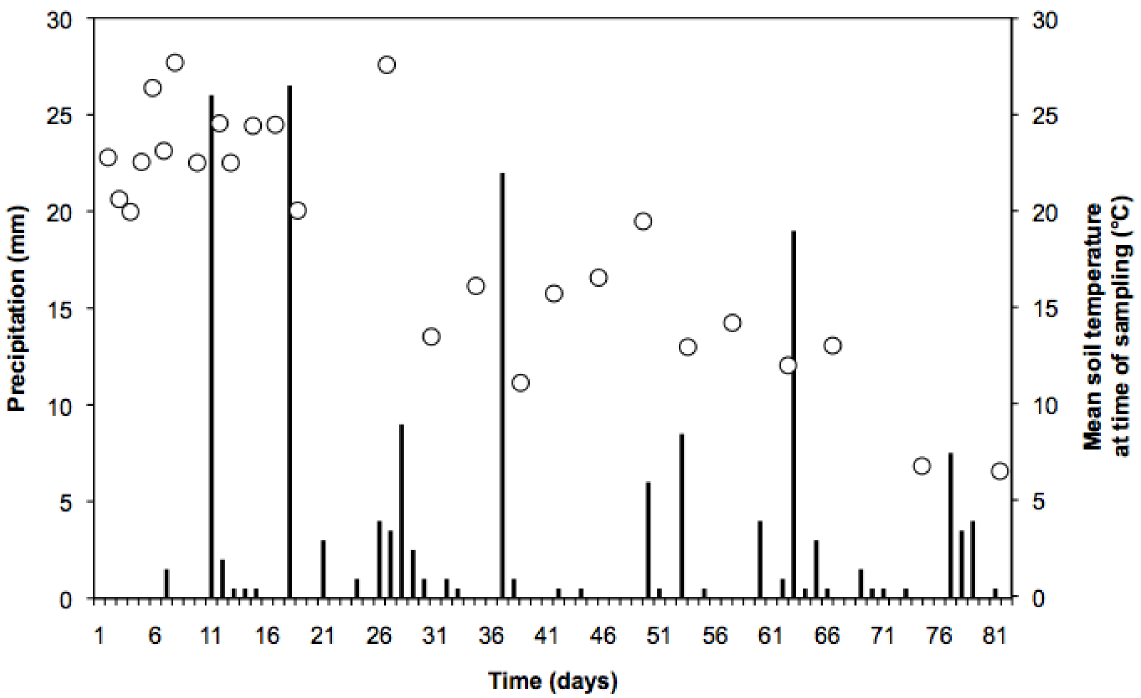

Supplementary Figure 2. Weather data during the experiment. Daily precipitation (bars) over the duration of experiment and mean soil temperature (open circles) on measurement days.

Supplementary Table 1. Values used and calculated in the illustrative example of the additive approach

**Source**	**Isotopic signature**	**Experiment 1**		**Experiment 2**	
		Fraction	Flux	Fraction	Flux
A	1.00	0.25	0.50	0.25	0.75
B	70.00	0.75	1.50	0.50	1.50
C	95.00			0.25	0.75
Total isotopic signature		**52.75**		**59.00**	

Supplementary Table 2. Isotopic values from this study (‰)

Parameter	Mean	Standard Error	N
$\delta_{Total-1}$	-27.09	0.47	7
$\delta_{Total-2}$	-25.33	0.30	7
δ_A (soil)	-29.31	0.30	7
δ_B (roots)	-13.37	0.01	5
δ_{C1} (PyOM-1)	37.46	5.30*	13
δ_{C2} (PyOM-2)	106.30	14.45*	13

*The high standard error on these numbers is likely an artifact of the small mass of sample required for PyOM C analyses (~2 mg). Because our experimental design required that we mix labelled and unlabelled (but otherwise identical) 350°C corn PyOM, it proved challenging to obtain a single 2-mg sample that is representative of the whole sample. Thus, we conducted many (13) analyses to be able to estimate their $\delta^{13}C$ values with confidence. However, this is likely an overestimate of the variance found in the actual experiment, because we weighed out the PyOM individually and precisely for each plot, and the sampling chamber included a much larger mass of PyOM.

Supplementary Table 3. Partitioned CO_2 fluxes (μmol CO_2 m^{-2} s^{-1}) including ("Baseline") or excluding ("No source σ^2") variation associated with all sources

Component	System	Scenario	Low $CI_{95\%}$	Mean CO_2	High $CI_{95\%}$
Soil	3-part	Baseline	1.04	1.25	1.47
		No source σ^2	1.00	1.25	1.50
	Soil+PyOM	Baseline	1.11	1.44	1.76
		No source σ^2	1.06	1.44	1.83
	Soil +Roots	Baseline	0.91	1.02	1.12
		No source σ^2	0.92	1.02	1.11
PyOM	3-part	Baseline	0.02	0.03	0.05
		No source σ^2	0.01	0.03	0.06
	Soil+PyOM	Baseline	0.04	0.06	0.08
		No source σ^2	0.04	0.05	0.07
Roots	3-part	Baseline	-0.08	0.04	0.17
		No source σ^2	-0.09	0.04	0.18
	Soil+Roots	Baseline	-0.02	0.03	0.08
		No source σ^2	0.00	0.03	0.06

Supplementary Table 4. Partitioned CO_2 fluxes under different source $\delta^{13}C$ values

Scenario	$\delta^{13}C$ value (‰)			CO_2 fluxes (µmol CO_2 m^{-2} s^{-1})		
	PyOM-1	PyOM-2	Roots	Soil	PyOM	Roots
Baseline	39.83	113.13	-13.37	1.252	0.034	0.041
Low PyOM	38.83	112.13	-13.37	1.249	0.034	0.044
High PyOM	40.83	114.13	-13.37	1.254	0.034	0.039
Baseline	39.83	113.13	-13.37	1.252	0.034	0.041
Low roots	39.83	113.13	-14.37	1.249	0.034	0.044
High roots	39.83	113.13	-12.37	1.254	0.034	0.039

Supplementary Table 5 Initial PyOM and corn stover properties

Property (units)	Value	
	PyOM	Stover
Total C (% w/w)	61.0	41.9
Total N (% w/w)	2.7	1.96
C:N (by mass)	22	21
Total H (% w/w)	3.9	
Total O (% w/w)	15	
pH_{DIW} (1:20 w/v)	10.0	
Feedstock	Corn	
Particle size (mm)	< 2	
Heating rate (°C min^{-1})	5	
Final temp (°C)	350	
Residence time (min)	45	
Surface area (m^2 g^{-1})	92.8	
ASTM Ash (%)	17	
ASTM Volatiles (%)	35	
ASTM Fixed C (%)	48	

Supplementary Table 6 Initial soil properties

Property (units)	Value
Texture	(Channery) silt loam
pH_{DIW}	6.0
Sand (%)	28.1
Silt (%)	54.7
Clay (%)	17.2
Total C (%)	1.48
Total N (%)	0.16
C:N (mass)	9.39

Supplementary Note 1. Comparing approaches to partitioning more than two sources

The approaches and their advantages and disadvantages are described in Table 1 of the main manuscript. While the additive approach and the modelling approach are both distinct, there are commonalities in the other four approaches – each changes the system in some way to create three equations, in order to solve for the three unknowns. We present the base equations here for each – note the similar format:

Multiple element approach:

(S1) $f_A + f_B + f_C = 1$

(S2) $\delta_{Total\text{-}element\text{-}1} = f_A * \delta_{A\text{-}element\ 1} + f_B * \delta_{B\text{-}element\ 1} + f_C * \delta_{C\text{-}element\ 1}$

(S3) $\delta_{Total\text{-}element\text{-}2} = f_A * \delta_{A\text{-}element\ 2} + f_B * \delta_{B\text{-}element\ 2} + f_C * \delta_{C\text{-}element\ 2}$

Here, the two elements could be, for example, ^{15}N and ^{13}C. The sources A, B, and C would ideally all have distinct isotopic signatures for each isotope, but this could also be designed similarly to the combined sources approach below, with mixed pairing of isotopes.

Multiple isotope approach:

(S4) $f_A + f_B + f_C = 1$

(S5) $\delta_{Total\text{-}isotope\text{-}1} = f_A * \delta_{A\text{-}isotope\ 1} + f_B * \delta_{B\text{-}isotope\ 1} + f_C * \delta_{C\text{-}isotope\ 1}$

(S6) $\delta_{Total\text{-}isotope\text{-}2} = f_A * \delta_{A\text{-}isotope\ 2} + f_B * \delta_{B\text{-}isotope\ 2} + f_C * \delta_{C\text{-}isotope\ 2}$

Here, the two isotopes considered could be, for example, ^{14}C and ^{13}C. Again, the sources A, B, and C would ideally all have distinct isotopic signatures for each isotope, but this could also be designed similarly to the combined sources approach below, with mixed pairing of isotopes.

Combined sources approach:

(S7) $f_A + f_B + f_C = 1$

(S8) $\delta_{Total\text{-}1} = (f_A + f_B) * \delta_{A\&B} + f_C * \delta_C$

(S9) $\delta_{Total\text{-}2} = f_A * \delta_A + (f_B + f_C) * \delta_{B\&C}$

Here, the experiment is designed such that components A and B have the same isotopic signature in one treatment, and B and C have the same signature in a second treatment. Similarly, A and C could also be paired, depending on how the sources are produced.

Paired treatments approach (this paper):

(S10) $f_A + f_B + f_C = 1$

(S11) $\delta_{Total\text{-}1} = f_A * \delta_A + f_B * \delta_B + f_C * \delta_{C1}$

(S12) $\delta_{Total\text{-}2} = f_A * \delta_A + f_B * \delta_B + f_C * \delta_{C2}$

Here, the experiment is designed so that all components, A, B, and C, have distinct signatures, and C has two different possible signatures.

The additive approach (Supplementary Figure 1): This approach does not mirror the others, but, rather, assumes that a component conclusively partitioned in a

separate treatment (e.g., PyOM and soil only, or roots and soil only) remains constant in the three-part treatment. This component is subtracted from the three-part treatment, and then the remaining C is partitioned between the two remaining sub-components. We provide a worked example here:

Sources A, B, and C, have the isotopic values $\delta_A = 1$, $\delta_B = 70$, and $\delta_C = 95$, respectively. We measure A and B in experiment 1, and A, B, and C in experiment 2 (Supplementary Figure 1).

In experiment 1, we measure a total flux of 2 μmol m^{-2} s^{-1} and a combined isotopic value of $\delta_{Total} = 52.75$. Using

(S13) $f_A = 1 - f_B$ and

(S14) $f_B = (\delta_{Total} – \delta_A) / (\delta_B - \delta_A)$, we solve to get

$f_B = 0.75$ and $f_A = 0.25$.

Thus, the flux from A is 2 μmol m^{-2} s^{-1} • 0.25 = 0.5 μmol m^{-2} s^{-1} and the flux from B is 2 μmol m^{-2} s^{-1} • 0.75 = 1.5 μmol m^{-2} s^{-1}.

In experiment 2, we measure a total flux of 3 μmol m^{-2} s^{-1} and a combined isotopic value of $\delta_{Total} = 59$. We could take a few approaches. The simplest would be to argue that the increase must all derive from C, since adding C increased total flux by 1 μmol m^{-2} s^{-1}. However, this makes it impossible to detect any interactions. We could improve the estimate, if we know something about the

system – say, for example, we think we can assume that the flux from B remained constant. We can simplify to:

(S15) $\delta_{Total} = f_A * \delta_A + f_B * \delta_B + f_C * \delta_C$

$59 = f_A * 1 + (1.5/3) * 70 + f_C * 95$

$24 = f_A * 1 + f_C * 95$

We can also simplify:

(S1) $f_A + f_B + f_C = 1$

$f_A + (1.5/3) + f_C = 1$

$f_A = 0.5 - f_C$.

Now the equations for this three-part system are in the same from as S7 and S8, and we can solve as above to find $f_A = 0.25$ and $f_C = 0.25$.

Thus, the flux from A is 3 µmol m^{-2} s^{-1} • 0.25 = 0.75 µmol m^{-2} s^{-1} and the flux from C is 3 µmol m^{-2} s^{-1} • 0.25 = 0.75 µmol m^{-2} s^{-1}. Since we held B constant, we assumed its flux is 1.5 µmol m^{-2} s^{-1}. Results are summarized in Supplementary Table 1.

Using this method, we were able to detect that there must be some interaction between A and C – when C was added to the system, the flux from A increased from 0.5 to 0.75 µmol m^{-2} s^{-1}. However, this approach required that we assumed B did not change between experiment 1 and experiment 2. Thus, we would be unable to detect if B changed in the three-part system.

Supplementary Note 2. Error propagation for equations (7-9)

To calculate the variance associated with each fraction, we show two approaches. First, we discuss the general Taylor series equation[1] as worked out below, where we estimated the variance associated with each fraction based on partial derivatives with respect to each parameter, and the variance of each parameter. Second, we discuss an approach where we calculated all possible solutions to the equation using matrices, from which we derived the distribution of the parameters in question. The error estimates are very similar, and we show the results from the second method in figure 2. We include R scripts for both of these approaches to solving the system of equations (7-9) and estimating variance as additional supplementary materials.

Taylor series equations

δ_X is the known isotopic ratio of the individual sources (δ_A, δ_B, δ_{C1}, and δ_{C2}) or their combined total (δ_{T1} and δ_{T2}). f_A, f_B, and f_C represent the fraction of the total pool made up by A, B, and C, respectively. σ^2_X is the variance associated with a given value, x.

For Equation 7 in the main manuscript, $f_C = (\delta_{T1} - \delta_{T2}) / (\delta_{C1} - \delta_{C2})$,

we calculate the partial derivatives for f_C with respect to each variable:

$$\frac{\partial f_C}{\partial \delta_{T1}} = \frac{1}{\delta_{C1} - \delta_{C2}}$$

$$\frac{\partial f_C}{\partial \delta_{T2}} = \frac{-1}{\delta_{C1} - \delta_{C2}}$$

$$\frac{\partial f_{\mathrm{C}}}{\partial \delta_{\mathrm{C1}}} = \frac{-(\delta_{\mathrm{T1}} - \delta_{\mathrm{T2}})}{(\delta_{\mathrm{C1}} - \delta_{\mathrm{C2}})^2}$$

$$\frac{\partial f_{\mathrm{C}}}{\partial \delta_{\mathrm{C2}}} = \frac{(\delta_{\mathrm{T1}} - \delta_{\mathrm{T2}})}{(\delta_{\mathrm{C1}} - \delta_{\mathrm{C2}})^2} \quad \text{(S16)}$$

We then use these derivatives to calculate the variance associated with f_{C}:

$$\sigma\delta_{f_{\mathrm{C}}}^2 = \left(\frac{\partial f_{\mathrm{C}}}{\partial \delta_{\mathrm{T1}}}\right)^2 \cdot \sigma_{\delta_{\mathrm{T1}}}^2 + \left(\frac{\partial f_{\mathrm{C}}}{\partial \delta_{\mathrm{T2}}}\right)^2 \cdot \sigma_{\delta_{\mathrm{T2}}}^2 + \left(\frac{\partial f_{\mathrm{C}}}{\partial \delta_{\mathrm{C1}}}\right)^2 \cdot \sigma_{\delta_{\mathrm{C1}}}^2 + \left(\frac{\partial f_{\mathrm{C}}}{\partial \delta_{\mathrm{C2}}}\right)^2 \cdot \sigma_{\delta_{\mathrm{C2}}}^2$$

$$\sigma_{f_{\mathrm{C}}}^2 = \left(\frac{1}{\delta_{\mathrm{C1}} - \delta_{\mathrm{C2}}}\right)^2 \cdot \sigma_{\delta_{\mathrm{T1}}}^2 + \left(\frac{-1}{\delta_{\mathrm{C1}} - \delta_{\mathrm{C2}}}\right)^2 \cdot \sigma_{\delta_{\mathrm{T2}}}^2 + \left(\frac{-(\delta_{\mathrm{T1}} - \delta_{\mathrm{T2}})}{(\delta_{\mathrm{C1}} - \delta_{\mathrm{C2}})^2}\right)^2 \cdot \sigma_{\delta_{\mathrm{C1}}}^2 + \left(\frac{(\delta_{\mathrm{T1}} - \delta_{\mathrm{T2}})}{(\delta_{\mathrm{C1}} - \delta_{\mathrm{C2}})^2}\right)^2 \cdot \sigma_{\delta_{\mathrm{C2}}}^2$$

which simplifies to:

$$\sigma_{f_{\mathrm{C}}}^2 = \frac{\left(\sigma_{\delta_{\mathrm{T1}}}^2 + \sigma_{\delta_{\mathrm{T2}}}^2\right)}{(\delta_{\mathrm{C1}} - \delta_{\mathrm{C2}})^2} + \frac{(\delta_{\mathrm{T1}} - \delta_{\mathrm{T2}})^2}{(\delta_{\mathrm{C1}} - \delta_{\mathrm{C2}})^4} \cdot \left(\sigma_{\delta_{\mathrm{C1}}}^2 + \sigma_{\delta_{\mathrm{C2}}}^2\right) \quad \text{(S17)}$$

For Equation 8.1, $f_{\mathrm{A}} = (\delta_{\mathrm{T1}} - \delta_{\mathrm{B}} + f_{\mathrm{C}}\,(\delta_{\mathrm{B}} - \delta_{\mathrm{C1}}))\, /\, (\delta_{\mathrm{A}} - \delta_{\mathrm{B}}))$, or

$f_{\mathrm{A}} = (\delta_{\mathrm{T1}} - \delta_{\mathrm{B}} + f_{\mathrm{C}}\,\delta_{\mathrm{B}} - f_{\mathrm{C}}\,\delta_{\mathrm{C1}}))\, /\, (\delta_{\mathrm{A}} - \delta_{\mathrm{B}})$,

we calculate the partial derivatives for f_{A} with respect to each variable:

$$\frac{\partial f_{\mathrm{A}}}{\partial \delta_{\mathrm{T1}}} = \frac{1}{\delta_{\mathrm{A}} - \delta_{\mathrm{B}}}$$

$$\frac{\partial f_{\mathrm{A}}}{\partial \delta_{\mathrm{B}}} = \frac{f_{\mathrm{C}}(\delta_{\mathrm{A}} - \delta_{\mathrm{C}}) + \delta_{\mathrm{T1}} - \delta_{\mathrm{A}}}{(\delta_{\mathrm{A}} - \delta_{\mathrm{B}})^2}$$

$$\frac{\partial f_{\mathrm{A}}}{\partial f_{\mathrm{C}}} = \frac{\delta_{\mathrm{B}} - \delta_{\mathrm{C1}}}{\delta_{\mathrm{A}} - \delta_{\mathrm{B}}}$$

$$\frac{\partial f_{\mathrm{A}}}{\partial \delta_{\mathrm{C1}}} = \frac{-f_{\mathrm{C}}}{\delta_{\mathrm{A}} - \delta_{\mathrm{B}}}$$

$$\frac{\partial f_{\mathrm{A}}}{\partial \delta_{\mathrm{A}}} = \frac{-(\delta_{\mathrm{T1}} - \delta_{\mathrm{B}} + f_{\mathrm{C}} \cdot \delta_{\mathrm{B}} - f_{\mathrm{C}} \cdot \delta_{\mathrm{C1}})}{(\delta_{\mathrm{A}} - \delta_{\mathrm{B}})^2} \quad \text{(S18)}$$

We then use these derivatives to calculate the variance associated with f_{A}:

$$\sigma_{f_A}^2 = \left(\frac{\partial f_A}{\partial \delta_{T1}}\right)^2 \cdot \sigma_{\delta_{T1}}^2 + \left(\frac{\partial f_A}{\partial \delta_B}\right)^2 \cdot \sigma_{\delta_B}^2 + \left(\frac{\partial f_A}{\partial f_C}\right)^2 \cdot \sigma_{f_C}^2 + \left(\frac{\partial f_A}{\partial \delta_{C1}}\right)^2 \cdot \sigma_{\delta_{C1}}^2 + \left(\frac{\partial f_A}{\partial \delta_A}\right)^2 \cdot \sigma_{\delta_A}^2$$

$$\sigma_{f_A}^2 = \left(\frac{1}{\delta_A - \delta_B}\right)^2 \cdot \sigma_{\delta_{T1}}^2 + \left(\frac{f_C(\delta_A - \delta_{C1}) + \delta_{T1} - \delta_A}{(\delta_A - \delta_B)^2}\right)^2 \cdot \sigma_{\delta_B}^2 + \left(\frac{\delta_B - \delta_{C1}}{\delta_A - \delta_B}\right)^2 \cdot \sigma_{f_C}^2 + \left(\frac{-f_C}{\delta_A - \delta_B}\right)^2 \cdot \sigma_{\delta_{C1}}^2 +$$

$$\left(\frac{-(\delta_{T1} - \delta_B + f_C \cdot \delta_B - f_C \cdot \delta_{C1})}{(\delta_A - \delta_B)^2}\right)^2 \cdot \sigma_{\delta_A}^2$$

which simplifies to:

$$\sigma_{f_{A1}}^2 = \frac{1}{(\delta_A - \delta_B)^2} \cdot \sigma_{\delta_{T1}}^2 + \frac{(f_C(\delta_A - \delta_{C1}) + \delta_{T1} - \delta_A)^2}{(\delta_A - \delta_B)^4} \cdot \sigma_{\delta_B}^2 + \frac{(\delta_B - \delta_{C1})^2}{(\delta_A - \delta_B)^2} \cdot \sigma_{f_C}^2 +$$

$$\frac{{f_C}^2}{(\delta_A - \delta_B)^2} \cdot \sigma_{\delta_{C1}}^2 + \frac{(\delta_{T1} - \delta_B + f_C \cdot \delta_B - f_C \cdot \delta_{C1})^2}{(\delta_A - \delta_B)^4} \cdot \sigma_{\delta_A}^2$$

$$\sigma_{f_A}^2 = \frac{\sigma_{\delta_{T1}}^2 + (\delta_B - \delta_{C1})^2 \cdot \sigma_{f_C}^2 + {f_C}^2 \cdot \sigma_{\delta_{C1}}^2}{(\delta_A - \delta_B)^2} + \frac{(f_C(\delta_A - \delta_{C1}) + \delta_{T1} - \delta_A)^2 \cdot \sigma_{\delta_B}^2 + (\delta_{T1} - \delta_B + f_C \cdot (\delta_B - \delta_{C1}))^2 \cdot \sigma_{\delta_A}^2}{(\delta_A - \delta_B)^4}$$

(S19.1)

Similarly, for Equation 8.2 in the main manuscript, we would obtain:

$$\sigma_{f_{A1}}^2 = \frac{\sigma_{\delta_{T2}}^2 + (\delta_B - \delta_{C2})^2 \cdot \sigma_{f_C}^2 + {f_C}^2 \cdot \sigma_{\delta_{C2}}^2}{(\delta_A - \delta_B)^2} + \frac{(f_C(\delta_A - \delta_{C2}) + \delta_{T2} - \delta_A)^2 \cdot \sigma_{\delta_B}^2 + (\delta_{T2} - \delta_B + f_C \cdot (\delta_B - \delta_{C2}))^2 \cdot \sigma_{\delta_A}^2}{(\delta_A - \delta_B)^4}$$

(S19.2)

Because we have two estimates for f_A, we take their mean, and their mean variance:

$$f_A = \frac{(f_{A1} + f_{A2})}{2}$$

$$\sigma_{f_{\mathrm{A}}}^2 = \frac{\left(\sigma_{f_{\mathrm{A1}}}^2 + \sigma_{f_{\mathrm{A2}}}^2\right)}{2}$$

(S19.3)

For Equation 9 in the main manuscript, $f_B = 1 - f_C - f_A$, we calculate the partial derivatives for f_B with respect to each variable:

$$\frac{\partial f_{\mathrm{B}}}{\partial f_{\mathrm{C}}} = -1$$

$$\frac{\partial f_{\mathrm{B}}}{\partial f_{\mathrm{A}}} = -1$$

We then use these derivatives to calculate the variance associated with f_B:

$$\sigma_{f_{\mathrm{B}}}^2 = \left(\frac{\partial f_{\mathrm{B}}}{\partial f_{\mathrm{C}}}\right)^2 \cdot \sigma_{f_{\mathrm{C}}}^2 + \left(\frac{\partial f_{\mathrm{B}}}{\partial f_{\mathrm{A}}}\right)^2 \cdot \sigma_{f_{\mathrm{A}}}^2$$

$$\sigma_{f_{\mathrm{B}}}^2 = (-1)^2 \cdot \sigma_{f_{\mathrm{C}}}^2 + (-1)^2 \cdot \sigma_{f_{\mathrm{A}}}^2$$

$$\sigma_{f_{\mathrm{B}}}^2 = \sigma_{f_{\mathrm{C}}}^2 + \sigma_{f_{\mathrm{A}}}^2 \text{ (S20)}$$

In order to calculate the variance associated with the fluxes (*F*), we take a similar approach:

$F_A = F_{Total} \cdot f_A$

$$\frac{\partial F_{\mathrm{A}}}{\partial f_{\mathrm{A}}} = F_{\mathrm{Total}}$$

$$\frac{\partial F_{\mathrm{A}}}{\partial F_{\mathrm{Total}}} = f_{\mathrm{A}}$$

$$\sigma_{F_{\mathrm{A}}}^2 = \left(\frac{\partial F_{\mathrm{A}}}{\partial F_{\mathrm{Total}}}\right)^2 \cdot \sigma_{F_{\mathrm{Total}}}^2 + \left(\frac{\partial F_{\mathrm{A}}}{\partial f_{\mathrm{A}}}\right)^2 \cdot \sigma_{f_{\mathrm{A}}}^2$$

$$\sigma_{F_{\mathrm{A}}}^2 = (f_{\mathrm{A}})^2 \cdot \sigma_{F_{\mathrm{Total}}}^2 + (F_{\mathrm{Total}})^2 \cdot \sigma_{f_{\mathrm{A}}}^2 \text{ (S21)}$$

Similarly,

$\sigma^2_{F_\mathrm{B}} = (f_\mathrm{B})^2 \cdot \sigma^2_{F_\mathrm{Total}} + (F_\mathrm{Total})^2 \cdot \sigma^2_{f_\mathrm{B}}$ (S22)

and

$\sigma^2_{F_\mathrm{C}} = (f_\mathrm{C})^2 \cdot \sigma^2_{F_\mathrm{Total}} + (F_\mathrm{Total})^2 \cdot \sigma^2_{f_\mathrm{C}}$ (S23).

For standard two-part partitioning, we will not work through the examples here in full, but note that for:

$f_\mathrm{A} = (\delta_\mathrm{Total} – \delta_\mathrm{B}) / (\delta_\mathrm{A} - \delta_\mathrm{B})$ and $f_\mathrm{B} = 1 - f_\mathrm{A}$,

$$\sigma^2_{f_\mathrm{A}} = \frac{\left(\sigma^2_{\delta_\mathrm{T}}\right)}{(\delta_\mathrm{A}-\delta_\mathrm{B})^2} + \frac{(\delta_\mathrm{T}-\delta_\mathrm{B})^2 \cdot \sigma^2_{\delta_\mathrm{A}} + (\delta_\mathrm{T}-\delta_\mathrm{A})^2 \cdot \sigma^2_{\delta_\mathrm{B}}}{(\delta_\mathrm{A}-\delta_\mathrm{B})^4} \text{ (S24)}$$

and

$\sigma^2_{f_\mathrm{B}} = \sigma^2_{f_\mathrm{A}}$ (S25).

Matrix solution of distributions

We arranged equations (7-9) in matrices as follows:

$$R = \begin{matrix} \delta_\mathrm{T1} \\ \delta_\mathrm{T2} \\ 1 \end{matrix}$$

$$D = \begin{matrix} \delta_\mathrm{A} & \delta_\mathrm{A} & 1 \\ \delta_\mathrm{B} & \delta_\mathrm{B} & 1 \\ \delta_\mathrm{C1} & \delta_\mathrm{C2} & 1 \end{matrix}$$

$$K = \begin{matrix} f_\mathrm{A} \\ f_\mathrm{B} \\ f_\mathrm{C} \end{matrix}$$

where δ_X is the known isotopic ratio of the individual sources (δ_A, δ_B, δ_C1, and δ_C2) or their combined total (δ_T1 and δ_T2). f_A, f_B, and f_C represent the fraction of the total pool made up by A, B, and C, respectively. σ^2_X is the variance associated with a given value, X.

We solve the system of equations for one complete set of measured values (δ_A, δ_B, δ_{C1}, δ_{C2}, δ_{T1} and δ_{T2}) by fitting the model

R = K D,

solving for the K coefficients f_A, f_B, and f_C, and multiplying them by the mean flux (F_{Mean}) for that specific set of values:

$F_A = f_A \bullet F_{Mean}$

$F_B = f_B \bullet F_{Mean}$

$F_C = f_C \bullet F_{Mean}$

We repeat this process for every possible combination of values, yielding a distribution of values for fluxes F_A, F_B, and F_C. From this distribution, we calculate the mean and standard error (taking care to use n=number of field replicates, not total possible combinations!) and calculate a 95% confidence interval. We interpret non-overlapping 95% confidence intervals as indicating significant differences at $p<0.05$.

Supplementary Note 3. Simulating effects of changing variance in total fluxes or isotopic ratios of sources

We investigated the effects of variance in the isotopic ratios of the sources on our partitioning (using the Taylor series approach described above). This variance could be due to heterogeneity within the sources, due to natural variability, or uneven isotopic label incorporation (Supplementary Table 2). We recalculated the partitioning without accounting for the variability for all the sources' isotopic ratios, and compared this to the baseline scenario (including source variance) (Supplementary Table 3). The data show that assuming the isotopic signatures had no variability had relatively little effect on our calculation of the size of the variance and the confidence intervals on the partitioned fluxes. It is clear that the majority of the variance in the system is due to the variability in the measured total fluxes and their associated isotopic values.

Supplementary Note 4. Possible effects of isotopic fractionation

We also investigate the effect of possible isotopic fractionation during the processes of PyOM mineralization and root C respiration. Because we were able to measure the δ^{13}C values of SOC mineralization in the field on its own, we are confident this is a good estimate. However, because it is challenging to measure root C respiration or mineralization of PyOM in isolation in the field (although it is possible to estimate in the lab – *e.g.*, Ref. 2), it is important to consider the possible effects of isotopic fractionation. We have previously measured isotopic fractionation in the mineralization of ^{13}C-labelled PyOM of about +0.5‰ (comparing CO_2 evolved in a 350°C sugar maple PyOM-sand incubation vs. bulk material) and fractionation in root respiration of corn (*Zea mays* L.) of less than about +1‰ (comparing CO_2 from roots in a "C-free" soil vs. bulk shoots or roots)[2]. However, it is also important to note that the δ^{13}C of mineralized PyOM has been observed to change over time[3,4], likely as different sub-fractions with slightly different δ^{13}C signatures are mineralized. While we will not investigate the effects of shifting δ^{13}C signatures over time, we report how the flux partitioning would differ under different δ^{13}C values for PyOM-C mineralization and for root respiration (Supplementary Note 4 and Supplementary Table 4).
Isotopic fractionation of ±1‰ in PyOM or roots would have a moderate effect on flux partitioning results, varying from <1% (SOC and PyOM) up to 7% (roots) (Supplementary Table 4). In this system, the greatest effects are observed on the determination of root C - because they make up a relatively small fraction of the

total, shifts in $\delta^{13}C$ affect them more. The contribution of PyOM does not shift, so long as the isotopic signatures from both PyOM sources shift equally, because its calculation (Equation 7 in the main manuscript) depends on the difference between the two sources. Thus, intriguingly, if fractionation affects the two sources similarly, we can expect relatively high confidence in the calculations for the contribution of PyOM.

Supplementary References

1. Phillips, D. L. & Gregg, J. W. Uncertainty in source partitioning using stable isotopes. *Oecologia* **127,** 171–179 (2001).
2. Whitman, T., Enders, A. & Lehmann, J. Pyrogenic carbon additions to soil counteract positive priming of soil carbon mineralization by plants. *Soil Biol. Biochem.* **73,** 33–41 (2014).
3. Zimmerman, A. R., Bin Gao & Ahn, M.-Y. Positive and negative carbon mineralization priming effects among a variety of biochar-amended soils. *Soil Biol. Biochem.* **43,** 1169–1179 (2011).
4. Whitman, T., Zhu, Z. & Lehmann, J. Carbon mineralizability determines interactive effects on mineralization of pyrogenic organic matter and soil organic carbon. *Environ. Sci. Technol.* **48,** 13727–13734 (2014).
